# 4,5,6-Tri-*O*-acetyl-2,3-di-*S*-ethyl-2,3-dithio-d-allose diethyl dithio­acetal

**DOI:** 10.1107/S1600536809015694

**Published:** 2009-05-07

**Authors:** Xiao-Dong Xi, Da-Xin Shi, Hui Li, Yun-Zheng Li, Qin-Pei Wu

**Affiliations:** a School of Chemical Engineering and Environment, Beijing Institute of Technology, Beijing 100081, People’s Republic of China; b School of Science, Beijing Institute of Technology, Beijing 100081, People’s Republic of China

## Abstract

The title compound, C_20_H_36_O_6_S_4_, was obtained by ethanethiol­ysis of 3,5,6-tri-*O*-acetyl-1,2-*O*-isopropyl­idene-α-d-gluco­furan­­ose. One of the ethyl groups is disordered over two sites with refined occupancies of 0.869 (6) and 0.131 (6). Compared with the precursor, the absolute configuration of the stereocenters at positions C-3 and C-2 are inverted and maintained, respectively.

## Related literature

For the bioactiviy of nucleosides, see: Zhang *et al.* (2007 ▶); Merino *et al.* (2008 ▶). For the structure of the precursor, 3,5,6-tri-*O*-acetyl-1,2-*O*-isopropyl­idene-α-d-glucofuran­ose, see: Wu *et al.* (2009 ▶). For related structures, see: Bethel & Ferrier (1972 ▶); Berrang & Hortor (1970 ▶); Divjaković *et al.* (1992 ▶).
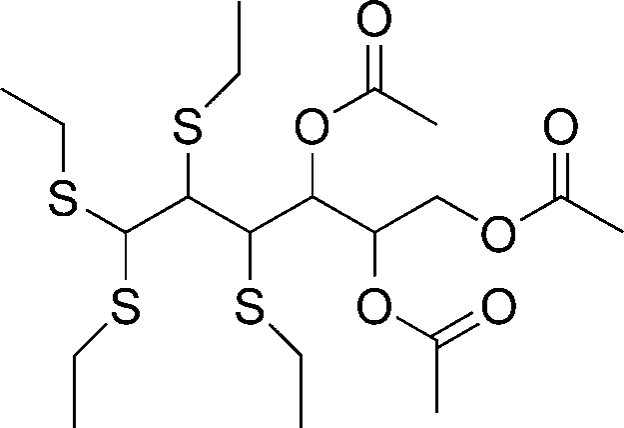


## Experimental

### 

#### Crystal data

C_20_H_36_O_6_S_4_
*M* *_r_* = 500.73
Monoclinic, 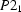

*a* = 8.3395 (12) Å
*b* = 16.726 (2) Å
*c* = 9.2027 (14) Å
β = 95.772 (5)°
*V* = 1277.2 (3) Å^3^
*Z* = 2
Mo *K*α radiation
μ = 0.40 mm^−1^
*T* = 113 K
0.26 × 0.20 × 0.18 mm

#### Data collection

Rigaku Saturn diffractometer
Absorption correction: multi-scan (*CrystalClear*; Rigaku/MSC, 2005 ▶) *T*_min_ = 0.902, *T*_max_ = 0.931
16052 measured reflections
6060 independent reflections
5802 reflections with *I* > 2σ(*I*)
*R*_int_ = 0.032

#### Refinement

*R*[*F* ^2^ > 2σ(*F* ^2^)] = 0.032
*wR*(*F* ^2^) = 0.066
*S* = 1.04
6060 reflections
298 parameters
23 restraints
H-atom parameters constrained
Δρ_max_ = 0.20 e Å^−3^
Δρ_min_ = −0.29 e Å^−3^
Absolute structure: Flack (1983 ▶), 2919 Friedel pairs
Flack parameter: −0.03 (4)

### 

Data collection: *CrystalClear* (Rigaku/MSC, 2005 ▶); cell refinement: *CrystalClear*; data reduction: *CrystalClear*; program(s) used to solve structure: *SHELXS97* (Sheldrick, 2008 ▶); program(s) used to refine structure: *SHELXL97* (Sheldrick, 2008 ▶); molecular graphics: *SHELXTL* (Sheldrick, 2008 ▶); software used to prepare material for publication: *SHELXTL*.

## Supplementary Material

Crystal structure: contains datablocks global, I. DOI: 10.1107/S1600536809015694/lh2804sup1.cif

Structure factors: contains datablocks I. DOI: 10.1107/S1600536809015694/lh2804Isup2.hkl

Additional supplementary materials:  crystallographic information; 3D view; checkCIF report

## Figures and Tables

**Fig. 1.**
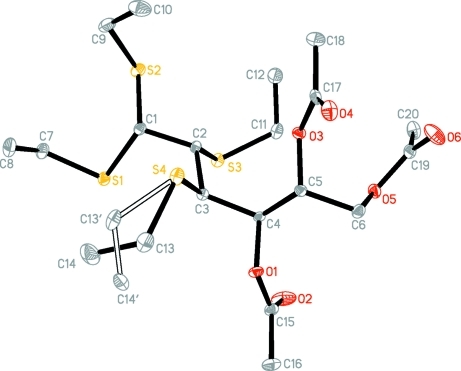
Molecular structure of the title compound with thermal displacement ellipsoids drawn at the 30% probability level. Open bonds show the disorder component.

## supplementary crystallographic information

### Comment

Nucleosides are important compounds due to their extensive bioactivity in antitumor and antivirus (Zhang *et al.*, 2007, Merino *et al.*, 2008). In the course of our studies in the synthesis of nucleoside analogues, an ethanethio substituted thioacetal product was obtained as ethanethiolysis of 3,5,6-tri-*O*-acetyl-1,2-*O*-isopropylidene-α-*D*-glucofuranose (Wu *et al.*, 2009). The purpose of the structure determination was to establish the position of the substituents of the ethanethio groups and the molecular conformation of the title compound. In contrast to its precursor, the absolute configuration of C-3 and C-2 are inverted and maintained, respectively, which is similar to the documented results (Berrang & Hortor, 1970; Bethel & Ferrier, 1972), but disagrees with the molecular structure reported by Divjaković (Divjakovic *et al.*, 1992). Both the molecular conformation and structure are consistent with our desired intermediates for the synthesis of the corresponding 2',3'-dideoxy-2',3'-dimercaptoribonucleosides.

### Experimental

ZnBr_2_ (0.54 g, 2.4 mmol) was added to a solution of 3,5,6-tri-*O*-acetyl-1,2-*O*-isopropylidene-α-D-glucofuranose (0.67 g, 2.0 mmol) and ethanethiol (0.78 ml, 10 mmol) in dry CH_2_Cl_2_ (10 ml). After 5 h, saturated aqueous solution of NaHCO_3_ (20 ml) was added and separated. The organic layer was washed with brine, dried with MgSO_4_ and concentrated under reduced pressure. The residue was isolated through short column chromatography on silica gel, which was eluded with EtOAc-petroleum to give the target compound (0.78 g, 78%). M. p. 62°, ^1^H-NMR(CDCl_3_, p.p.m.): 1.28 (m, 12 H), 2.06 (m, 9 H), 2.75 (m, 8 H), 3.07 (dd, 1 H), 3.48 (dd, 1 H), 4.20 (m, 1 H), 4.49 (dd, 1 H), 4.6 4 (d, 1 H), 5.54 (m, 1 H), 5.89 (dd, 1 H); ^13^C-NMR(CDCl_3_, p.p.m.):14.60, 14.74, 14.85, 14.92, 15.02, 21.23, 21.27, 25.79, 26.39, 28.31, 30.24, 51.47, 57.30, 57.48, 62.56, 71.96, 72.78, 169.19, 169.61, 170.82; HRMS (EI) m/*z* calculated for C_20_H_36_O_6_S_4_ 500.13, found 500.14.

50 mg of the obtained product was dissolved in petroleum ether (5 ml) and the solution was kept at room temperature for 2 days to give colorless single crystals.

### Refinement

C—H were included in the riding model approximation with C—H distances 0.98–1.00 Å, and with *U*_iso_=1.2*U*_eq_(C) or 1.5*U*_eq_(C_methyl_).

One of the ethyl groups is disordered over two sites with refined occupancies of 0.869 (6) and 0.131 (6). The restained distance of C13—C14 refined to 1.513 (4)Å and C13'-C14' refined to 1.502 (19) Å.

### Crystal data

**Table:**

C_20_H_36_O_6_S_4_	*F*(000) = 536
*M**_r_* = 500.73	*D*_x_ = 1.302 Mg m^−^^3^
Monoclinic, *P*2_1_	Mo *K*α radiation, λ = 0.71070 Å
Hall symbol: P 2yb	Cell parameters from 4760 reflections
*a* = 8.3395 (12) Å	θ = 2.2–27.9°
*b* = 16.726 (2) Å	µ = 0.40 mm^−^^1^
*c* = 9.2027 (14) Å	*T* = 113 K
β = 95.772 (5)°	Block, colorless
*V* = 1277.2 (3) Å^3^	0.26 × 0.20 × 0.18 mm
*Z* = 2	

### Data collection

**Table:**

Rigaku Saturn diffractometer	6060 independent reflections
Radiation source: rotating anode	5802 reflections with *I* > 2σ(*I*)
confocal	*R*_int_ = 0.032
Detector resolution: 14.63 pixels mm^-1^	θ_max_ = 27.9°, θ_min_ = 2.2°
ω scans	*h* = −10→10
Absorption correction: multi-scan (*CrystalClear*; Rigaku/MSC, 2005)	*k* = −21→22
*T*_min_ = 0.902, *T*_max_ = 0.931	*l* = −12→12
16052 measured reflections	

### Refinement

**Table:**

Refinement on *F*^2^	Secondary atom site location: difference Fourier map
Least-squares matrix: full	Hydrogen site location: inferred from neighbouring sites
*R*[*F*^2^ > 2σ(*F*^2^)] = 0.032	H-atom parameters constrained
*wR*(*F*^2^) = 0.066	*w* = 1/[σ^2^(*F*_o_^2^) + (0.0326*P*)^2^] where *P* = (*F*_o_^2^ + 2*F*_c_^2^)/3
*S* = 1.04	(Δ/σ)_max_ = 0.001
6060 reflections	Δρ_max_ = 0.20 e Å^−^^3^
298 parameters	Δρ_min_ = −0.29 e Å^−^^3^
23 restraints	Absolute structure: Flack (1983), 2919 Friedel pairs
Primary atom site location: structure-invariant direct methods	Flack parameter: −0.03 (4)

### Special details

**Table:**

Geometry. All e.s.d.'s (except the e.s.d. in the dihedral angle between two l.s. planes) are estimated using the full covariance matrix. The cell e.s.d.'s are taken into account individually in the estimation of e.s.d.'s in distances, angles and torsion angles; correlations between e.s.d.'s in cell parameters are only used when they are defined by crystal symmetry. An approximate (isotropic) treatment of cell e.s.d.'s is used for estimating e.s.d.'s involving l.s. planes.
Refinement. Refinement of *F*^2^ against ALL reflections. The weighted *R*-factor *wR* and goodness of fit *S* are based on *F*^2^, conventional *R*-factors *R* are based on *F*, with *F* set to zero for negative *F*^2^. The threshold expression of *F*^2^ > σ(*F*^2^) is used only for calculating *R*-factors(gt) *etc*. and is not relevant to the choice of reflections for refinement. *R*-factors based on *F*^2^ are statistically about twice as large as those based on *F*, and *R*- factors based on ALL data will be even larger.

### Fractional atomic coordinates and isotropic or equivalent isotropic displacement parameters (Å^2^)

**Table:**

	*x*	*y*	*z*	*U*_iso_*/*U*_eq_	Occ. (<1)
S1	0.74081 (5)	−0.08494 (3)	0.79166 (5)	0.02103 (10)	
S2	0.70511 (5)	0.08746 (3)	0.88989 (5)	0.02203 (10)	
S3	0.41930 (5)	0.00547 (3)	0.66199 (5)	0.01940 (10)	
S4	0.85901 (5)	0.02564 (3)	0.42590 (5)	0.02148 (10)	
O1	0.56270 (15)	−0.06561 (7)	0.23706 (13)	0.0182 (3)	
O2	0.32000 (18)	−0.12020 (10)	0.25917 (18)	0.0405 (4)	
O3	0.54225 (14)	0.14186 (7)	0.33931 (12)	0.0169 (3)	
O4	0.68402 (15)	0.21225 (8)	0.18563 (14)	0.0269 (3)	
O5	0.25647 (13)	0.10093 (8)	0.19236 (13)	0.0209 (3)	
O6	0.26208 (18)	0.22570 (8)	0.10243 (18)	0.0378 (4)	
C1	0.74322 (19)	0.02080 (11)	0.74033 (17)	0.0171 (3)	
H1	0.8521	0.0336	0.7099	0.021*	
C2	0.61765 (18)	0.03465 (11)	0.60845 (18)	0.0156 (3)	
H2	0.6143	0.0933	0.5873	0.019*	
C3	0.66042 (18)	−0.00888 (10)	0.46908 (17)	0.0147 (3)	
H3	0.6708	−0.0670	0.4933	0.018*	
C4	0.53286 (19)	−0.00125 (11)	0.33657 (17)	0.0148 (3)	
H4	0.4224	−0.0068	0.3689	0.018*	
C5	0.5413 (2)	0.07353 (10)	0.24304 (18)	0.0164 (4)	
H5	0.6440	0.0727	0.1957	0.020*	
C6	0.3999 (2)	0.08169 (12)	0.12490 (18)	0.0202 (4)	
H6A	0.4227	0.1244	0.0554	0.024*	
H6B	0.3843	0.0309	0.0700	0.024*	
C7	0.8968 (2)	−0.08772 (14)	0.94224 (19)	0.0284 (4)	
H7A	0.8763	−0.0444	1.0112	0.034*	
H7B	0.8888	−0.1391	0.9944	0.034*	
C8	1.0684 (2)	−0.07889 (15)	0.9010 (3)	0.0398 (5)	
H8A	1.0926	−0.1230	0.8366	0.060*	
H8B	1.1441	−0.0801	0.9896	0.060*	
H8C	1.0788	−0.0279	0.8503	0.060*	
C9	0.8906 (2)	0.14400 (14)	0.9124 (2)	0.0311 (5)	
H9A	0.8924	0.1759	1.0031	0.037*	
H9B	0.9821	0.1061	0.9247	0.037*	
C10	0.9154 (3)	0.19954 (14)	0.7876 (3)	0.0395 (5)	
H10A	0.9222	0.1683	0.6984	0.059*	
H10B	1.0155	0.2297	0.8104	0.059*	
H10C	0.8245	0.2368	0.7729	0.059*	
C11	0.2932 (2)	0.08823 (13)	0.59307 (19)	0.0237 (4)	
H11A	0.3106	0.0975	0.4896	0.028*	
H11B	0.1789	0.0730	0.5962	0.028*	
C12	0.3242 (2)	0.16570 (13)	0.6765 (2)	0.0302 (5)	
H12A	0.2985	0.1585	0.7773	0.045*	
H12B	0.2561	0.2080	0.6296	0.045*	
H12C	0.4378	0.1806	0.6766	0.045*	
C13	0.9482 (3)	−0.06248 (16)	0.3541 (3)	0.0317 (8)	0.869 (6)
H13A	0.8677	−0.0883	0.2826	0.038*	0.869 (6)
H13B	1.0406	−0.0461	0.3013	0.038*	0.869 (6)
C14	1.0059 (4)	−0.1230 (2)	0.4699 (3)	0.0428 (9)	0.869 (6)
H14A	1.0905	−0.0991	0.5378	0.064*	0.869 (6)
H14B	1.0490	−0.1700	0.4233	0.064*	0.869 (6)
H14C	0.9154	−0.1392	0.5235	0.064*	0.869 (6)
C13'	0.9867 (15)	−0.0625 (8)	0.4615 (17)	0.024 (5)	0.131 (6)
H13C	1.0967	−0.0488	0.4379	0.029*	0.131 (6)
H13D	0.9937	−0.0741	0.5674	0.029*	0.131 (6)
C14'	0.935 (2)	−0.1377 (8)	0.381 (2)	0.036 (4)	0.131 (6)
H14D	0.8573	−0.1665	0.4339	0.054*	0.131 (6)
H14E	1.0297	−0.1717	0.3722	0.054*	0.131 (6)
H14F	0.8855	−0.1240	0.2827	0.054*	0.131 (6)
C15	0.4443 (2)	−0.12014 (11)	0.20382 (19)	0.0200 (4)	
C16	0.4899 (3)	−0.17588 (12)	0.0885 (2)	0.0277 (4)	
H16A	0.4155	−0.2214	0.0805	0.042*	
H16B	0.6000	−0.1952	0.1143	0.042*	
H16C	0.4843	−0.1477	−0.0053	0.042*	
C17	0.6157 (2)	0.20907 (11)	0.2941 (2)	0.0209 (4)	
C18	0.5959 (3)	0.27634 (12)	0.3979 (2)	0.0326 (5)	
H18A	0.6485	0.3244	0.3644	0.049*	
H18B	0.6454	0.2617	0.4954	0.049*	
H18C	0.4810	0.2871	0.4021	0.049*	
C19	0.2065 (2)	0.17773 (11)	0.1783 (2)	0.0221 (4)	
C20	0.0766 (2)	0.19526 (13)	0.2743 (2)	0.0301 (5)	
H20A	0.1246	0.2033	0.3748	0.045*	
H20B	0.0012	0.1502	0.2712	0.045*	
H20C	0.0187	0.2437	0.2397	0.045*	

### Atomic displacement parameters (Å^2^)

**Table:**

	*U*^11^	*U*^22^	*U*^33^	*U*^12^	*U*^13^	*U*^23^
S1	0.0232 (2)	0.0209 (2)	0.0183 (2)	−0.00083 (19)	−0.00179 (18)	0.00364 (18)
S2	0.0236 (2)	0.0275 (2)	0.0155 (2)	−0.00207 (19)	0.00384 (17)	−0.00323 (19)
S3	0.0155 (2)	0.0251 (2)	0.0183 (2)	−0.00173 (18)	0.00489 (16)	0.00091 (18)
S4	0.01363 (19)	0.0294 (3)	0.0218 (2)	−0.00129 (18)	0.00362 (16)	0.00257 (19)
O1	0.0183 (6)	0.0201 (7)	0.0166 (6)	−0.0007 (5)	0.0034 (5)	−0.0072 (5)
O2	0.0312 (8)	0.0411 (9)	0.0523 (10)	−0.0176 (7)	0.0194 (7)	−0.0234 (8)
O3	0.0213 (6)	0.0138 (6)	0.0157 (6)	−0.0013 (5)	0.0026 (5)	0.0002 (5)
O4	0.0221 (7)	0.0292 (8)	0.0298 (7)	−0.0023 (6)	0.0050 (6)	0.0102 (6)
O5	0.0175 (6)	0.0194 (7)	0.0253 (7)	0.0012 (5)	−0.0005 (5)	0.0021 (5)
O6	0.0368 (8)	0.0244 (8)	0.0556 (10)	0.0038 (6)	0.0209 (8)	0.0118 (7)
C1	0.0174 (8)	0.0212 (9)	0.0128 (8)	−0.0003 (7)	0.0018 (6)	−0.0010 (7)
C2	0.0128 (8)	0.0171 (8)	0.0167 (8)	−0.0016 (7)	0.0006 (6)	−0.0001 (6)
C3	0.0146 (8)	0.0165 (9)	0.0133 (8)	0.0000 (7)	0.0028 (6)	0.0000 (7)
C4	0.0153 (8)	0.0159 (8)	0.0132 (8)	0.0026 (7)	0.0009 (6)	−0.0056 (7)
C5	0.0152 (8)	0.0166 (9)	0.0172 (9)	0.0016 (7)	0.0016 (6)	−0.0023 (7)
C6	0.0209 (9)	0.0257 (10)	0.0138 (8)	0.0023 (8)	0.0011 (7)	−0.0024 (7)
C7	0.0352 (10)	0.0305 (11)	0.0176 (9)	0.0017 (9)	−0.0064 (8)	0.0041 (9)
C8	0.0276 (11)	0.0400 (13)	0.0482 (14)	0.0029 (10)	−0.0139 (9)	0.0012 (11)
C9	0.0263 (10)	0.0344 (12)	0.0317 (11)	−0.0028 (9)	−0.0008 (8)	−0.0138 (9)
C10	0.0338 (11)	0.0283 (12)	0.0586 (15)	−0.0064 (10)	0.0160 (11)	−0.0079 (11)
C11	0.0174 (8)	0.0356 (11)	0.0182 (9)	0.0039 (8)	0.0029 (7)	0.0010 (9)
C12	0.0293 (11)	0.0346 (12)	0.0271 (11)	0.0066 (9)	0.0041 (8)	−0.0036 (9)
C13	0.0213 (12)	0.0515 (18)	0.0234 (15)	0.0072 (11)	0.0073 (10)	−0.0077 (11)
C14	0.0442 (17)	0.046 (2)	0.0393 (18)	0.0195 (14)	0.0118 (13)	−0.0025 (14)
C13'	0.007 (7)	0.045 (12)	0.022 (10)	−0.005 (6)	0.004 (6)	0.002 (7)
C14'	0.032 (7)	0.024 (7)	0.050 (8)	0.010 (6)	−0.009 (6)	−0.005 (6)
C15	0.0265 (10)	0.0197 (9)	0.0138 (9)	−0.0023 (8)	0.0025 (7)	−0.0008 (7)
C16	0.0377 (11)	0.0240 (10)	0.0222 (10)	−0.0048 (9)	0.0067 (8)	−0.0076 (8)
C17	0.0155 (8)	0.0178 (9)	0.0281 (10)	0.0003 (7)	−0.0037 (7)	0.0074 (8)
C18	0.0386 (12)	0.0200 (10)	0.0382 (13)	−0.0062 (9)	−0.0003 (10)	−0.0018 (8)
C19	0.0166 (8)	0.0230 (10)	0.0253 (10)	0.0003 (8)	−0.0043 (7)	0.0008 (8)
C20	0.0242 (10)	0.0332 (12)	0.0329 (12)	0.0057 (9)	0.0033 (8)	0.0084 (9)

### Geometric parameters (Å, °)

**Table:**

S1—C7	1.8035 (18)	C9—C10	1.507 (3)
S1—C1	1.8313 (19)	C9—H9A	0.9900
S2—C9	1.807 (2)	C9—H9B	0.9900
S2—C1	1.8238 (17)	C10—H10A	0.9800
S3—C11	1.814 (2)	C10—H10B	0.9800
S3—C2	1.8387 (16)	C10—H10C	0.9800
S4—C13	1.806 (2)	C11—C12	1.515 (3)
S4—C13'	1.829 (13)	C11—H11A	0.9900
S4—C3	1.8348 (16)	C11—H11B	0.9900
O1—C15	1.357 (2)	C12—H12A	0.9800
O1—C4	1.4510 (19)	C12—H12B	0.9800
O2—C15	1.200 (2)	C12—H12C	0.9800
O3—C17	1.365 (2)	C13—C14	1.513 (4)
O3—C5	1.446 (2)	C13—H13A	0.9900
O4—C17	1.199 (2)	C13—H13B	0.9900
O5—C19	1.352 (2)	C14—H14A	0.9800
O5—C6	1.439 (2)	C14—H14B	0.9800
O6—C19	1.188 (2)	C14—H14C	0.9800
C1—C2	1.539 (2)	C13'—C14'	1.502 (19)
C1—H1	1.0000	C13'—H13C	0.9900
C2—C3	1.547 (2)	C13'—H13D	0.9900
C2—H2	1.0000	C14'—H14D	0.9800
C3—C4	1.541 (2)	C14'—H14E	0.9800
C3—H3	1.0000	C14'—H14F	0.9800
C4—C5	1.524 (2)	C15—C16	1.491 (2)
C4—H4	1.0000	C16—H16A	0.9800
C5—C6	1.527 (2)	C16—H16B	0.9800
C5—H5	1.0000	C16—H16C	0.9800
C6—H6A	0.9900	C17—C18	1.496 (3)
C6—H6B	0.9900	C18—H18A	0.9800
C7—C8	1.524 (3)	C18—H18B	0.9800
C7—H7A	0.9900	C18—H18C	0.9800
C7—H7B	0.9900	C19—C20	1.495 (3)
C8—H8A	0.9800	C20—H20A	0.9800
C8—H8B	0.9800	C20—H20B	0.9800
C8—H8C	0.9800	C20—H20C	0.9800
			
C7—S1—C1	101.38 (9)	C9—C10—H10A	109.5
C9—S2—C1	101.20 (9)	C9—C10—H10B	109.5
C11—S3—C2	102.08 (8)	H10A—C10—H10B	109.5
C13—S4—C13'	32.2 (5)	C9—C10—H10C	109.5
C13—S4—C3	103.66 (10)	H10A—C10—H10C	109.5
C13'—S4—C3	103.3 (4)	H10B—C10—H10C	109.5
C15—O1—C4	118.30 (13)	C12—C11—S3	114.35 (13)
C17—O3—C5	116.06 (13)	C12—C11—H11A	108.7
C19—O5—C6	115.63 (14)	S3—C11—H11A	108.7
C2—C1—S2	110.29 (11)	C12—C11—H11B	108.7
C2—C1—S1	108.94 (11)	S3—C11—H11B	108.7
S2—C1—S1	112.86 (9)	H11A—C11—H11B	107.6
C2—C1—H1	108.2	C11—C12—H12A	109.5
S2—C1—H1	108.2	C11—C12—H12B	109.5
S1—C1—H1	108.2	H12A—C12—H12B	109.5
C1—C2—C3	112.81 (13)	C11—C12—H12C	109.5
C1—C2—S3	107.93 (11)	H12A—C12—H12C	109.5
C3—C2—S3	112.69 (11)	H12B—C12—H12C	109.5
C1—C2—H2	107.7	C14—C13—S4	113.70 (19)
C3—C2—H2	107.7	C14—C13—H13A	108.8
S3—C2—H2	107.7	S4—C13—H13A	108.8
C4—C3—C2	114.75 (13)	C14—C13—H13B	108.8
C4—C3—S4	111.65 (11)	S4—C13—H13B	108.8
C2—C3—S4	108.65 (11)	H13A—C13—H13B	107.7
C4—C3—H3	107.1	C14'—C13'—S4	117.0 (11)
C2—C3—H3	107.1	C14'—C13'—H13C	108.0
S4—C3—H3	107.1	S4—C13'—H13C	108.0
O1—C4—C5	103.41 (12)	C14'—C13'—H13D	108.0
O1—C4—C3	106.83 (13)	S4—C13'—H13D	108.0
C5—C4—C3	116.60 (14)	H13C—C13'—H13D	107.3
O1—C4—H4	109.9	C13'—C14'—H14D	109.5
C5—C4—H4	109.9	C13'—C14'—H14E	109.5
C3—C4—H4	109.9	H14D—C14'—H14E	109.5
O3—C5—C4	107.49 (13)	C13'—C14'—H14F	109.5
O3—C5—C6	108.79 (13)	H14D—C14'—H14F	109.5
C4—C5—C6	113.55 (14)	H14E—C14'—H14F	109.5
O3—C5—H5	109.0	O2—C15—O1	123.03 (17)
C4—C5—H5	109.0	O2—C15—C16	126.52 (18)
C6—C5—H5	109.0	O1—C15—C16	110.42 (15)
O5—C6—C5	109.18 (13)	C15—C16—H16A	109.5
O5—C6—H6A	109.8	C15—C16—H16B	109.5
C5—C6—H6A	109.8	H16A—C16—H16B	109.5
O5—C6—H6B	109.8	C15—C16—H16C	109.5
C5—C6—H6B	109.8	H16A—C16—H16C	109.5
H6A—C6—H6B	108.3	H16B—C16—H16C	109.5
C8—C7—S1	115.40 (14)	O4—C17—O3	123.77 (18)
C8—C7—H7A	108.4	O4—C17—C18	126.18 (18)
S1—C7—H7A	108.4	O3—C17—C18	110.05 (15)
C8—C7—H7B	108.4	C17—C18—H18A	109.5
S1—C7—H7B	108.4	C17—C18—H18B	109.5
H7A—C7—H7B	107.5	H18A—C18—H18B	109.5
C7—C8—H8A	109.5	C17—C18—H18C	109.5
C7—C8—H8B	109.5	H18A—C18—H18C	109.5
H8A—C8—H8B	109.5	H18B—C18—H18C	109.5
C7—C8—H8C	109.5	O6—C19—O5	124.29 (17)
H8A—C8—H8C	109.5	O6—C19—C20	124.31 (18)
H8B—C8—H8C	109.5	O5—C19—C20	111.37 (16)
C10—C9—S2	114.64 (15)	C19—C20—H20A	109.5
C10—C9—H9A	108.6	C19—C20—H20B	109.5
S2—C9—H9A	108.6	H20A—C20—H20B	109.5
C10—C9—H9B	108.6	C19—C20—H20C	109.5
S2—C9—H9B	108.6	H20A—C20—H20C	109.5
H9A—C9—H9B	107.6	H20B—C20—H20C	109.5
			
C9—S2—C1—C2	−119.35 (13)	S4—C3—C4—C5	−39.69 (17)
C9—S2—C1—S1	118.55 (11)	C17—O3—C5—C4	153.04 (14)
C7—S1—C1—C2	178.77 (11)	C17—O3—C5—C6	−83.62 (17)
C7—S1—C1—S2	−58.37 (11)	O1—C4—C5—O3	−169.73 (12)
S2—C1—C2—C3	169.79 (11)	C3—C4—C5—O3	−52.85 (18)
S1—C1—C2—C3	−65.83 (15)	O1—C4—C5—C6	69.89 (16)
S2—C1—C2—S3	−65.04 (13)	C3—C4—C5—C6	−173.23 (13)
S1—C1—C2—S3	59.34 (13)	C19—O5—C6—C5	104.90 (17)
C11—S3—C2—C1	134.39 (12)	O3—C5—C6—O5	−48.42 (19)
C11—S3—C2—C3	−100.37 (12)	C4—C5—C6—O5	71.22 (18)
C1—C2—C3—C4	176.29 (14)	C1—S1—C7—C8	−71.74 (18)
S3—C2—C3—C4	53.74 (17)	C1—S2—C9—C10	68.51 (17)
C1—C2—C3—S4	−57.96 (16)	C2—S3—C11—C12	−71.05 (15)
S3—C2—C3—S4	179.49 (9)	C13'—S4—C13—C14	18.1 (8)
C13—S4—C3—C4	−87.95 (15)	C3—S4—C13—C14	−75.1 (2)
C13'—S4—C3—C4	−121.1 (5)	C13—S4—C13'—C14'	−38.2 (11)
C13—S4—C3—C2	144.51 (13)	C3—S4—C13'—C14'	56.4 (13)
C13'—S4—C3—C2	111.4 (5)	C4—O1—C15—O2	−4.4 (3)
C15—O1—C4—C5	−116.96 (15)	C4—O1—C15—C16	173.80 (15)
C15—O1—C4—C3	119.46 (15)	C5—O3—C17—O4	−3.9 (2)
C2—C3—C4—O1	−160.52 (13)	C5—O3—C17—C18	175.50 (14)
S4—C3—C4—O1	75.30 (15)	C6—O5—C19—O6	8.6 (3)
C2—C3—C4—C5	84.49 (17)	C6—O5—C19—C20	−169.42 (14)

## References

[bb1] Berrang, B. & Hortor, D. (1970). *Chem. Commun.* pp. 1038–1039.

[bb2] Bethel, G. S. & Ferrier, R. J. (1972). *J. Chem. Soc. Perkin Trans. 1*, pp. 1033–1037.

[bb3] Divjaković, V., Miljković, D., Lajšić, S. & Klement, U. (1992). *Acta Cryst.* C**48**, 1685–1686.

[bb4] Flack, H. D. (1983). *Acta Cryst.* A**39**, 876–881.

[bb5] Merino, P., Tejero, T. & Delso, I. (2008). *Curr. Med. Chem.* **15**, 954–967. 10.2174/092986708784049612 18393853

[bb6] Rigaku/MSC (2005). *CrystalClear* Rigaku/MSC Inc., The Woodlands, Texas, USA.

[bb7] Sheldrick, G. M. (2008). *Acta Cryst.* A**64**, 112–122. 10.1107/S0108767307043930 18156677

[bb8] Wu, Q.-P., Xi, X.-D., Li, H. & Zhang, Q.-Sh. (2009). *Chin. J. Chem.* In the press.

[bb9] Zhang, J., Visser, F., King, K. M., Baldwin, S. A., Young, J. D. & Cass, C. E. (2007). *Cancer Metastasis Rev.* **26**, 85–110. 10.1007/s10555-007-9044-4 17345146

